# The Effect of the Weight of Equipment on Muscle Activity of the Lower Extremity in Soldiers

**DOI:** 10.1100/2012/976513

**Published:** 2012-09-02

**Authors:** Tobias Lindner, Christoph Schulze, Sandra Woitge, Susanne Finze, Wolfram Mittelmeier, Rainer Bader

**Affiliations:** ^1^Department of Orthopaedics, University Medicine Rostock, Doberaner Straße 142, 18057 Rostock, Germany; ^2^Bundeswehr Institute of Sports Medicine, Dr.-Rau-Allee 32, 48231 Warendorf, Germany; ^3^Rostock Military Medical Centre, Hohe Düne 30, 18119 Rostock, Germany

## Abstract

Due to their profession and the tasks it entails, soldiers are exposed to high levels of physical activity and strain. This can result in overexertion and pain in the locomotor system, partly caused by carrying items of equipment. The aim of this study was to analyse the extent of muscle activity in the lower extremities caused by carrying specific items of equipment. For this purpose, the activity of selected groups of muscles caused by different items of equipment (helmet, carrying strap, backpack, and rifle) in the upper and lower leg was measured by recording dynamic surface electromyograms. Electrogoniometers were also used to measure the angle of the knee over the entire gait cycle. In addition to measuring muscle activity, the study also aimed to determine out what influence increasing weight load has on the range of motion (ROM) of the knee joint during walking. The activity of recorded muscles of the lower extremity, that is, the tibialis anterior, peroneus longus, gastrocnemius lateralis, gastrocnemius medialis, rectus femoris, and biceps femoris, was found to depend on the weight of the items of equipment. There was no evidence, however, that items of equipment weighing a maximum of 34% of their carrier's body weight had an effect on the ROM of the knee joint.

## 1. Introduction

Due to the high level of physical strain to which they are exposed and the specific physical tasks they are required to perform, soldiers run an increased risk of sustaining injuries, including overexertion injuries, to the locomotor system [1–3]. In this context, various predisposing factors such as personal fitness level, age, sex, smoking behaviour, or biomechanical characteristics such as the shape of the foot or spinal curvature play an important role [1, 2, 4–7]. One of the main causes of symptoms and injuries is strain resulting from carrying various items of equipment over long distances [3, 4]. Electromyographic tests on how backpack weight affects various muscles [8–11] have been carried out, as have tests on the kinematic and kinetic effects of equipment items [9, 12–15]. These tests reveal, for example, that the weight of equipment is significant for step length, step frequency, range of joint movement, and the orientation of the body axes in space [14].

In previous studies, it has also been established that the weight of equipment items also influences the activity of the trunk muscles [8, 16]. Knapik et al. [17] found that load-bearing systems that are supported on the hips influence the activity of the trapezius and erector spinae muscles. Schulze et al. [18] showed that soldiers' footwear can cause specific changes in the muscle activity of the lower extremity. Increased strain on lower extremity muscles is closely linked to the development of exertion-related symptoms, for example, shin splints or patellofemoral pain syndrome, which have a higher than average occurrence in soldiers [1]. In this context, there is a direct link between modified activity of the tibialis anterior muscle and the development of shin splints, whereas the activity of the rectus femoris muscle is of significance in connection with the development of functional knee pain [19, 20]. Modified muscular activity of the gastrocnemius lateralis muscle, in combination with impaired movement in the knee joint, negatively promotes the development of Achilles tendinopathies [21].

The aim of this study was to demonstrate, by means of electromyography, the effect of a successive increase in strain produced by specific items of equipment (helmet, carrying strap, backpack, and rifle) on the activity of selected muscle groups in the lower extremity, that is, the tibialis anterior, peroneus longus, gastrocnemius lateralis, gastrocnemius medialis, rectus femoris, and biceps femoris muscles, during walking. In addition, a goniometer was used to determine whether strain produced by equipment items changes the range of motion (ROM) of the knee joint during the gait cycle.

## 2. Materials and Methods

### 2.1. Participants

Thirty-seven German Air Force soldiers participated in this study on a voluntary basis. Five soldiers did not complete the analysis; the data obtained prior to them leaving the cohort was included in the evaluation. The participants were aged between 20 and 53 years (mean age: 29 years; median: 26 years). Their weight was between 62.5 and 112.0 kg (mean weight: 81.5 kg; median weight: 81.0 kg), their height between 163 and 193 cm (mean height: 177.8 cm; median: 179.0 cm), and their body mass index (BMI) between 21 and 34 kg/m^2^ (mean: 25.9 kg/m^2^; median: 26.0 kg/m^2^). All participants had completed their initial training and had been declared fit for duty when they participated in the study. Prior to the actual study recruitment, the soldiers underwent a physical examination to detect orthopaedic diseases and ROM of the ankle joint, knee, hip, and shoulder. We also examined the curvature of the spinal columns. The study protocol was approved by the Ethical Committee of the University of Rostock (file number: A 2009 36). All participants were fully informed about the content of the study and gave their written consent.

### 2.2. Equipment

**Figure 1:**
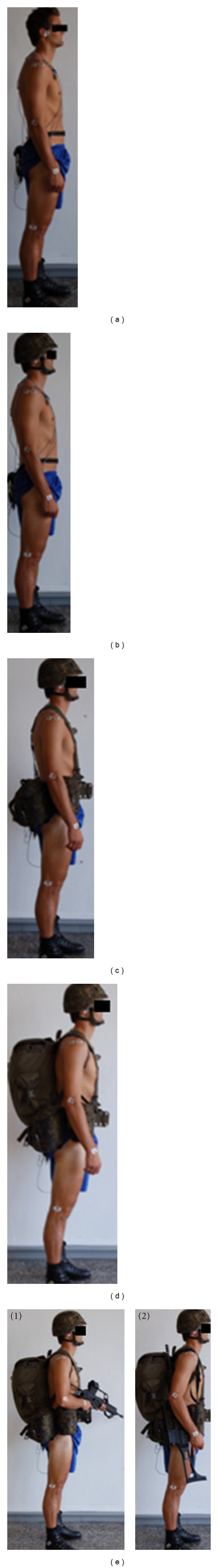
Equipment in order of investigation: (a) reference, (b) helmet, (c) load-carrying strap, (d) backpack, (e-1) rifle (in front of the body), and (e-2) rifle (slung over the right shoulder).

In the first test conditions (reference) the participants wore shorts, standard combat boots, and socks. In the order shown in Figure 1, all the participants wore the standard equipment of a soldier, consisting of a helmet, carrying strap, backpack, and rifle, consecutively. The weight of each item of additional equipment is specified in Table 1.

### 2.3. Instruments

Dynamic surface electromyograms (EMGs) of the peroneus longus, gastrocnemius lateralis, gastrocnemius medialis, tibialis anterior, rectus femoris, and biceps femoris muscles of the right leg were taken in accordance with the “standards for reporting EMG data” [22] of the International Society of Electrophysiology and Kinesiology using a wireless EMG system (Noraxon Telemyo 2400T, Noraxon, Scotsdale, Arizona, USA). Bipolar recordings were made, using disposable, self-adhesive Ag/AgCl electrodes (Blue Sensor P, Ambu, Germany) with an active electrode of diameter 7 mm. Before positioning the electrodes, their specified locations on the skin of each soldier were shaved, slightly roughened, cleaned with an alcohol pad, and air-dried. The electrodes were then placed both longitudinally and axially over the muscle belly of interest at a distance of approximately 40 mm (centre-to-centre) [23]. The EMG data were sampled at a frequency of 1500 Hz. The signals were amplified, filtered (10–400 Hz) and transmitted to a personal computer via a wireless transmitter.

In addition, the participants were equipped with an uniaxial electrogonimeter (Noraxon, Scotsdale, Arizona, USA) placed across the lateral side of the left knee to record flexion and extension angles for each gait cycle. All walking exercises took place on a standard motor-driven treadmill (Tempest, Kettler, Ense-Parsit, Germany) equipped with an inbuilt velocity/speed control. Surface EMG recordings were taken at each loading setup while the subjects were walking on the treadmill at a constant speed of 0.89 m/s (3.2 km/h).

### 2.4. Procedures

Following a two-minute warm-up walk on the treadmill at full test pace, EMG recordings were started with the first equipment setup. Muscle activity and knee angle were recorded using the EMG system. Each recording consisted of at least five double steps. After each loading setup, the participants added a piece of equipment in the order described in Figure 1. The warm-up period and the EMG, together with the knee angle recordings, were then restarted.

### 2.5. Data Processing

The software MyoResearch XP (Noraxon, Scotsdale, Arizona, USA) was used for subsequent processing of the EMG data. To determine the magnitude of muscle activity, the EMG data for each muscle was fully wave-rectified and smoothed by applying the root mean square calculation (RMS-EMG). The amount of muscle activity was determined by calculating mean EMG amplitude, peak, and area under the EMG curve (AUC or iEMG). All EMG data were normalised to the EMG measurement of the reference condition (a).

### 2.6. Statistical Analysis

Descriptive statistics (median, standard deviation, minimum, and maximum) were calculated for each dataset. After the Friedman test rejected the hypothesis of equality of the means for EMG mean amplitude, peak, and integral for different load conditions, the Wilcoxon test for pairwise comparison against control setup was performed. For this reason, it is important to mention that the measurements were dependent on the particular test subject. All *P* values are the result of two-tailed statistical tests, with values of *P* < 0.05 regarded as significant. All data were stored and analysed using the statistics program Statistical Package for Social Sciences for Windows (SPSS) Version 15.0 (SPSS Inc. Chicago, Illinois, USA).

## 3. Results

### 3.1. Electromyography

Analysis of the EMG data clearly revealed that the equipment items used influenced the activity of the muscles under examination. Mean amplitude, peak, and area under the curve (AUC) changed to varying degrees as a result of carrying different equipment items. Generally, relatively light items (helmet, carrying strap, and rifle)—in contrast to heavy items such as a backpack—caused little change in activity in the relevant muscles under observation.

**Figure 2:**
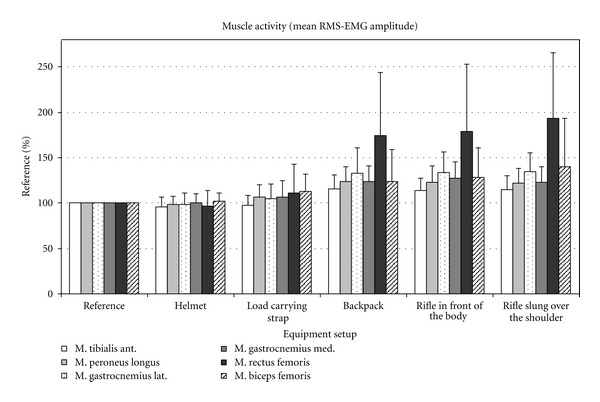
Mean EMG amplitude in percent compared to the mean EMG of the reference measurements.

**Figure 3:**
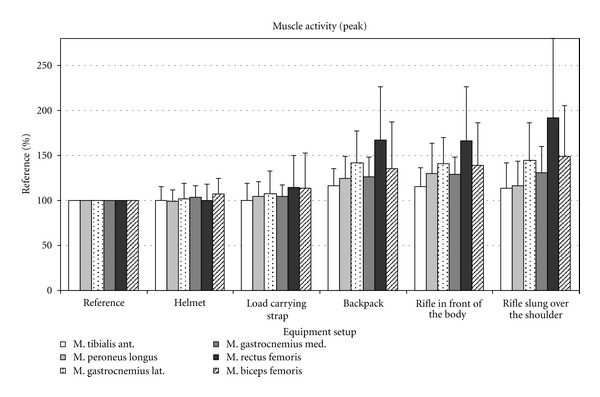
Peak EMG in percent compared to the peak EMG values of the reference measurements.

**Figure 4:**
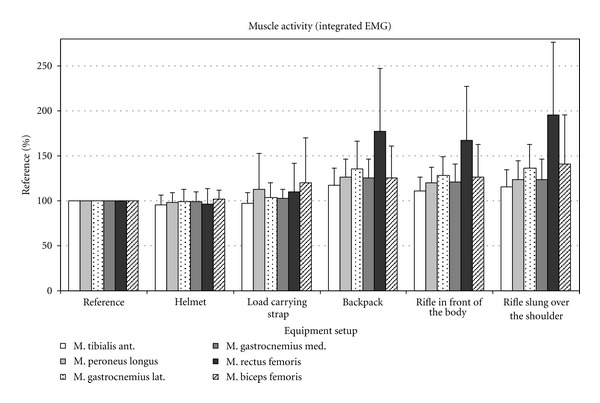
Integrated EMG in percent compared to the mean integrated EMG of the reference measurements.

Figures 2, 3, and 4 show the changes in EMG amplitude, EMG maximum and integrated EMG signal of the various muscles, depending on the various equipment items.

### 3.2. The Tibialis Anterior Muscle

When wearing a helmet, the mean (4%) and AUC EMG value (4.2%) of the tibialis anterior muscle (TA) fell slightly. In comparison to the reference measurement, the peak value remained the same. After adding the load-carrying strap, the activity of the TA changed very little (mean, peak, and integral). When the backpack was added, activity increased significantly for all three evaluated EMG parameters by approximately 16% in comparison to the control. Additionally, carrying the weapon, be it in front of the body or over the shoulder, had no influence on the activity of the TA muscle.

### 3.3. The Peroneus Longus Muscle

The treadmill analysis showed, in comparison to the reference value (*P* < 0.001), a significant increase in the activity of the peroneus longus (PL) muscle under an increasingly heavy equipment load when carrying the backpack. The helmet and load-carrying strap, on the other hand, did not cause any significant increase in activity in comparison to the reference value (Table 2). However, there was a significant increase in activity between the helmet and carrying strap load levels. Carrying the weapon, both in front of the body and slung over the shoulder, caused no significant differences in comparison to the backpack, except for a significantly different AUC value between the rucksack and carrying the weapon in front of the body (weapon e-1) and between the peak EMG values of the weapon when carried in front of the body or slung over the shoulder.

### 3.4. The Gastrocnemius Lateralis and Gastrocnemius Medialis Muscles

The two muscles, gastrocnemius laterialis (GL) and medialis (GM), did not show any significant increase in activity in comparison to the activity in the reference measurement when the subjects were wearing the helmet and carrying strap. Only when carrying the backpack was there a significant difference in comparison to the reference value for the mean, peak, and integral values. The activity of the GL and GM increased by 32% or 24% (both mean) in comparison to the reference value (*P* < 0.001).

As with the TA and PL muscles, there was no significant change in activity in the GL and GM muscles in comparison to the initial state when the weapon was added.

### 3.5. The Rectus Femoris Muscle

The slight decrease in activity in the rectus femoris muscle (RF) when the subjects were wearing the helmet was insignificant in comparison to the reference value. The slight increase in activity after adding the carrying strap, however, was significant both in comparison to the reference value (mean EMG *P* = 0.023) and in comparison to the helmet load level (*P* < 0.001). After the backpack was added, activity in the RF muscle increased by 75% (mean EMG) or 76% (AUC) in comparison to the value of the initial state (*P* < 0.001). Again, the change in muscle activity caused by adding the weapon, regardless of how it was carried, was not significant in comparison to the load level values of the backpack load level.

### 3.6. The Biceps Femoris Muscle

Similarly to the RF, there was a significant increase (mean and AUC) in the activity of the biceps femoris muscle (BF) after the carrying strap was added (*P* = 0.001, *P* = 0.002). The changes in the peak value for the BF were not significant in comparison to the initial state. In addition, significant muscle activity differences were revealed between carrying strap and backpack load levels (mean EMG *P* = 0.045 and AUC *P* = 0.021). As was the case for all muscles examined, there was no further increase in activity in the BF when the weapon was added.

### 3.7. Knee Angle Measurements

When the subjects were carrying the various items of equipment, mean values of the range of motion of the knee joint were between min. 55.1°  ± 8.2° (weapon carried in front of the body) and 56.8°  ± 6.6° (load-carrying strap), that is no significant differences were detected between the examined load levels.

## 4. Discussion

Military service places high demands on the physical fitness of soldiers. They frequently have to bear weighted loads and items of equipment during their everyday professional life. This is a potential risk factor for the occurrence of overexertion syndromes in the locomotor system [24, 25], particularly if the weight is carried over long distances. The aim of this study was to analyse the activity of selected muscles in the lower extremity on the basis of increasing weight caused by typical equipment items worn or carried by soldiers, for example helmet, carrying strap, backpack, and rifle. Al-Khabbaz et al. [26] showed that backpack weights of up to 20% of the carrier's bodyweight do not cause an increase in muscle activity in EMG measurements while standing. Simpson et al. [27], on the other hand, used measurements taken while subjects were walking to determine that there is an increase in activity (integrated EMG) of the vastus lateralis and gastrocnemius medialis muscles in female recreational walkers as a result of carrying a backpack weighing between 20 and 40% of their body weight. In our study, the muscles under examination (TA, PL, GL, GM, BF, and RF) showed the greatest increase in activity after adding the backpack. The greatest increase in muscle activity at this level of equipment load was detected in the rectus femoris muscle. This muscle plays a major role in the stretching of the knee [28]. Due to the relatively high additional weight of the backpack, which is between 15 and 30% of the personal body weight of the soldiers under examination, muscle activity also increased considerably as a result of the heavy load. A possible consequence of this increase in activity in soldiers can be frequent occurrence of functional knee pain [20]. The EMG changes in the gastrocnemius lateralis, gastrocnemius medialis, and tibialis anterior muscles revealed in this study could be indicative of the development of overexertion syndromes in the Achilles tendon and around the edge of the shinbone, because there is a link between a change in activity in the aforementioned muscles and the development of these symptoms [19, 21].

Due to its relatively low weight, the helmet showed no measurable influence on the muscle activity of the lower extremity, with the exception of the tibialis anterior muscle. A major proportion of the weight of the helmet is carried by the local muscles of the neck and upper back and leads to measurable differences in muscle activity in that area. Thuresson et al. [29] verified these increases in activity by means of EMG measurements of the neck muscles in helicopter pilots.

The effect of carrying a rifle on lower extremity activity has not been examined in previous EMG studies. Birrell and Haslam [24] examined the effect of carrying a rifle on ground reaction forces while walking, and discovered that effect to be significant. Our results showed that carrying a weapon exerts no additional influence on the activity of the examined muscles of the lower extremity. While the weight of the weapon is carried by the upper extremity, thus influencing the kinematics of the subjects' gait [24], the weapon, which in itself weighs 3.6 kg, has only a relatively low additional weight load—as is the case with a helmet and carrying strap—on muscular activity in the lower extremity.

In studies dealing with exertion-dependent changes in the movement range of the knee joint caused by the weight of equipment items [9, 12, 14, 15, 30], results have been inconsistent. While Attwells et al. [14] und Kinoshita et al. [12] observed greater ROM in the knee joint with increasing load, Ghori and Luckwill [9] found a decrease in ROM under load. Other authors, for example Majumdar et al. [15] or Tilbury-Davis and Hooper [30] did not observe any load-dependent change in the ROM of the knee joint. Our results also did not show any increase or decrease in the ROM in the various load situations. Majumdar et al. [15] claimed that the reason for the lack of differences is that the additional weight of between 6.5 and 27.2% of the subjects' body weight was too low. Tilbury-Davis and Hooper [30] presumed that the differences in Kinoshita's findings [24] can be attributed to the subjects being in better training condition and therefore having greater strength. With greater loads of between 47% and 64% of their body weight, these subjects were also able to maintain a normal gait pattern.

### 4.1. Limitations of the Study

In the examinations conducted, it must be borne in mind that this was a dynamic study and that gait phase-specific differences were also taken into account [31]. In dynamic studies, the centre of gravity is deflected in a sinusoidal movement in the transversal and sagittal planes [31]. The maximum is always reached in the middle standing phase on the side of the standing leg [31]. In the event of an unevenly distributed increasing load, appropriate stabilisation work becomes necessary. Thus stabilisation work, which increases in conjunction with the load, is also a component of the measured muscle activity and cannot be distinguished from the muscle activity that is brought about by changing the loads.

## 5. Conclusions

The equipment items used in our study are essential for soldiers to carry during military operations. For this reason, their influence on the activity of different muscles in the lower extremity was examined. By adding equipment items consecutively, we determined that relatively light items (helmet, carrying strap, and rifle) caused only minor changes in muscle activity. In contrast, heavy items such a backpack cause a considerable change in activity in the relevant muscles under observation. In our studies, the backpack, which weighed 15 kg, caused a mean 75% increase in muscle activity in comparison with the reference measurement. The loads that soldiers have to carry during marches should therefore be kept as low as possible given the possible reduction in the risk of musculoskeletal disorders.

## Figures and Tables

**Table 1:** Weight of different equipment items used in the measurements.

Equipment setup	Individual weight of the piece of equipment, kg	Weight of the equipment in total, kg	Setup number
Without equipment in shorts, combat boots and socks	—	—	a (Reference)
+ Helmet	1.5	1.5	b
+ Carrying strap	1	2.5	c
+ Backpack	15	17.5	d
+ Weapon G36			
(a) Carried in front of the body	3.6	21.1	e-1
(b) Slung over the shoulder	3.6	21.1	e-2

**Table 2:** Summary of significant effects on the EMG of different muscles.

Setup	EMG	Reference (a)			Helmet (b)			Carrying strap (c)			Backpack (d)			Weapon (e-1)			Weapon (e-2)		
		Mean	peak	AUC	Mean	peak	AUC	Mean	peak	AUC	Mean	peak	AUC	Mean	peak	AUC	Mean	peak	AUC
Muscle																			
	TA																		
	PL																		
Reference (a)	GL																		
	GM																		
	RF																		
	BF																		

	TA	∗	°	∗															
	PL	°	°	°															
Helmet (b)	GL	°	°	°															
	GM	°	°	°															
	RF	°	°	°															
	BF	°	°	°															

	TA	°	°	°	°	°	°												
	PL	°	°	°	∗∗∗	∗	∗∗												
Carrying strap (c)	GL	°	°	°	∗	°	°												
	GM	°	∗	°	∗	°	∗∗∗												
	RF	∗	∗	∗	∗∗∗	∗∗	∗∗∗												
	BF	∗∗	°	∗∗	∗∗∗	°	∗∗∗												

	TA	∗∗∗	∗∗∗	∗∗∗	∗∗∗	∗∗∗	∗∗∗	∗∗∗	∗∗∗	∗∗∗									
	PL	∗∗∗	∗∗∗	∗∗∗	∗∗∗	∗∗∗	∗∗∗	∗∗	∗∗	∗∗									
Backpack (d)	GL	∗∗∗	∗∗∗	∗∗∗	∗∗∗	∗∗∗	∗∗∗	∗∗∗	∗∗∗	∗∗∗									
	GM	∗∗∗	∗∗∗	∗∗∗	∗∗∗	∗∗∗	∗∗∗	∗∗∗	∗∗∗	∗∗∗									
	RF	∗∗∗	∗∗∗	∗∗∗	∗∗∗	∗∗∗	∗∗∗	∗∗∗	∗∗∗	∗∗∗									
	BF	∗∗	∗∗	∗∗	∗∗	∗	∗∗	∗	∗	∗									

	TA	∗∗∗	∗∗∗	∗∗∗	∗∗∗	∗∗∗	∗∗∗	∗∗∗	∗∗∗	∗∗∗	°	°	°						
	PL	∗∗∗	∗∗∗	∗∗∗	∗∗∗	∗∗∗	∗∗∗	∗∗	∗∗	∗∗	°	°	∗						
Weapon (e-1)	GL	∗∗∗	∗∗∗	∗∗∗	∗∗∗	∗∗∗	∗∗∗	∗∗∗	∗∗∗	∗∗∗	°	°	°						
	GM	∗∗∗	∗∗∗	∗∗∗	∗∗∗	∗∗∗	∗∗∗	∗∗∗	∗∗∗	∗∗∗	°	°	°						
	RF	∗∗∗	∗∗∗	∗∗∗	∗∗∗	∗∗∗	∗∗∗	∗∗∗	∗∗∗	∗∗∗	°	°	°						
	BF	∗∗∗	∗∗∗	∗∗	∗∗	∗∗	∗∗	∗∗	∗∗	∗	°	°	°						

	TA	∗∗∗	∗∗∗	∗∗∗	∗∗∗	∗	∗∗∗	∗∗∗	∗	∗∗∗	°	°	°	°	°	°			
	PL	∗∗∗	∗∗∗	∗∗∗	∗∗∗	∗∗∗	∗∗∗	∗	°	∗∗	°	°	°	°	∗	°			
Weapon (e-2)	GL	∗∗∗	∗∗∗	∗∗∗	∗∗∗	∗∗∗	∗∗∗	∗∗∗	∗∗∗	∗∗∗	°	°	°	°	°	°			
	GM	∗∗∗	∗∗∗	∗∗∗	∗∗∗	∗∗∗	∗	∗∗∗	∗∗∗	∗∗∗	°	°	°	°	°	°			
	RF	∗∗∗	∗∗∗	∗∗∗	∗∗∗	∗∗∗	∗∗∗	∗∗∗	∗∗∗	∗∗∗	°	°	°	°	°	°			
	BF	∗∗∗	∗∗	∗∗∗	∗∗∗	∗∗	∗∗∗	∗	∗	∗∗	∗	°	°	°	°	°			

°*P* > 0.05, *P < 0.05, **P < 0.01, and ***P < 0.001.

TA: tibialis anterior; BF: biceps femoris; PL: peroneus longus; RF: rectus femoris; GL: gastrocnemius lateralis; GM: gastrocnemius medialis.
